# Neurodegeneration and Neuroinflammation in Parkinson’s Disease: a Self-Sustained Loop

**DOI:** 10.1007/s11910-022-01207-5

**Published:** 2022-06-08

**Authors:** G. Arena, K. Sharma, G. Agyeah, R. Krüger, A. Grünewald, J. C. Fitzgerald

**Affiliations:** 1 Luxembourg Center for Systems Biomedicine (LCSB), University of Luxembourg, Esch-sur-Alzette, Luxembourg; 2 Department of Neurodegenerative Diseases, Hertie Institute for Clinical Brain Research, University of Tübingen, Tübingen, Germany; 3 Transversal Translational Medicine, Luxembourg Institute of Health, Strassen, Luxembourg; 4 Centre Hospitalier de Luxembourg (CHL), Luxembourg, Luxembourg; 5 Institute of Neurogenetics, University of Lübeck, Lübeck, Germany

**Keywords:** Parkinson’s disease, Inflammation, Gut microbiome, Protein aggregation, Mitochondrial dysfunction

## Abstract

**Purpose of Review:**

Neuroinflammation plays a significant role in Parkinson’s disease (PD) etiology along with mitochondrial dysfunction and impaired proteostasis. In this context, mechanisms related to immune response can act as modifiers at different steps of the neurodegenerative process and justify the growing interest in anti-inflammatory agents as potential disease-modifying treatments in PD. The discovery of inherited gene mutations in PD has allowed researchers to develop cellular and animal models to study the mechanisms of the underlying biology, but the original cause of neuroinflammation in PD is still debated to date.

**Recent Findings:**

Cell autonomous alterations in neuronal cells, including mitochondrial damage and protein aggregation, could play a role, but recent findings also highlighted the importance of intercellular communication at both local and systemic level. This has given rise to debate about the role of non-neuronal cells in PD and reignited intense research into the gut-brain axis and other non-neuronal interactions in the development of the disease. Whatever the original trigger of neuroinflammation in PD, what appears quite clear is that the aberrant activation of glial cells and other components of the immune system creates a vicious circle in which neurodegeneration and neuroinflammation nourish each other.

**Summary:**

In this review, we will provide an up-to-date summary of the main cellular alterations underlying neuroinflammation in PD, including those induced by environmental factors (e.g. the gut microbiome) and those related to the genetic background of affected patients. Starting from the lesson provided by familial forms of PD, we will discuss pathophysiological mechanisms linked to inflammation that could also play a role in idiopathic forms. Finally, we will comment on the potential clinical translatability of immunobiomarkers identified in PD patient cohorts and provide an update on current therapeutic strategies aimed at overcoming or preventing inflammation in PD.

## Introduction

Parkinson’s disease (PD) is characterized by the cardinal signs of the movement disorder such as tremor and postural instability, as well as the histopathological hallmarks of alpha-synuclein (α-syn) accumulation and loss of dopaminergic neurons in the *substantia nigra* (SN). Aggregated α-syn is the main component of Lewy bodies, which are proteinaceous neuronal inclusions found in the SN of PD brains [1, 2]. PD is clinically, pathologically and genetically heterogeneous, and the absence of LBs in some genetic cases has led to an ongoing discussion about the overlap between PD, parkinsonism and other neurodegenerative diseases. Other brain pathologies in PD include vascular pathology [3, 4], gliosis [5*•*] and brain lesions [6, 7].

Finding causative treatments for PD is a major challenge because the underlying pathobiology is complex, multifactorial and may differ from person to person. This has led researchers to explore whether there are common or converging pathways that could be exploited for pharmacological intervention (e.g. kinase activity, protein aggregation and inflammation). But there are further challenges; most of the dopaminergic neurons in the SN are often already lost at the point of clinical presentation, and therefore a lot of effort is being put into early disease detection and reliable biomarkers. Finally, many known PD pathomechanisms do not specifically affect the dopaminergic neurons of the SN and in some cases—including inflammation—are associated with a plethora of other diseases such as dementia, irritable bowel syndrome (IBS) and diabetes [8–10]. Today, dopamine replacement is still the mainstay treatment for PD, and the disease prevalence is drastically rising [11]. Finding causative treatments may reduce the disease prevalence or improve prognosis. Identification of markers at different stages of disease progression in different patient subtypes would help in predicting causative agents and drastically help in the search for a treatment strategy.

In the following sections, we will summarize current mechanistic concepts on the role of inflammation in PD. Based on the evidence provided by monogenic forms, we will first review the main cellular dysfunctions and molecular determinants that locally trigger CNS inflammation; moreover, we will examine more closely the pathophysiological mechanisms and metabolites that control peripheral dysbiosis-induced inflammation and their contribution to the development of PD. Finally, we will discuss potential controversies and provide future directions with a focus on the emerging fields, biomarkers and therapeutic strategies.

## Inflammation in PD

Sustained inflammatory processes are largely recognized as major patho-etiological mechanisms underlying neurodegeneration [12]. In contrast to acute inflammation, which is usually beneficial and contributes to the immediate repair of brain tissues exposed to various environmental insults (e.g. traumatic injury, viral infection, toxins), chronic inflammation is typically associated to the development and progression of neurodegenerative disorders [13]. Whether there is a trigger for neuroinflammation in PD is not fully understood, but conditions such as α-syn misfolding [14, 15], polymorphisms in immune-related genes [16, 17] and mitochondrial dysfunction [18, 19] have been suggested.

**Fig. 1.**
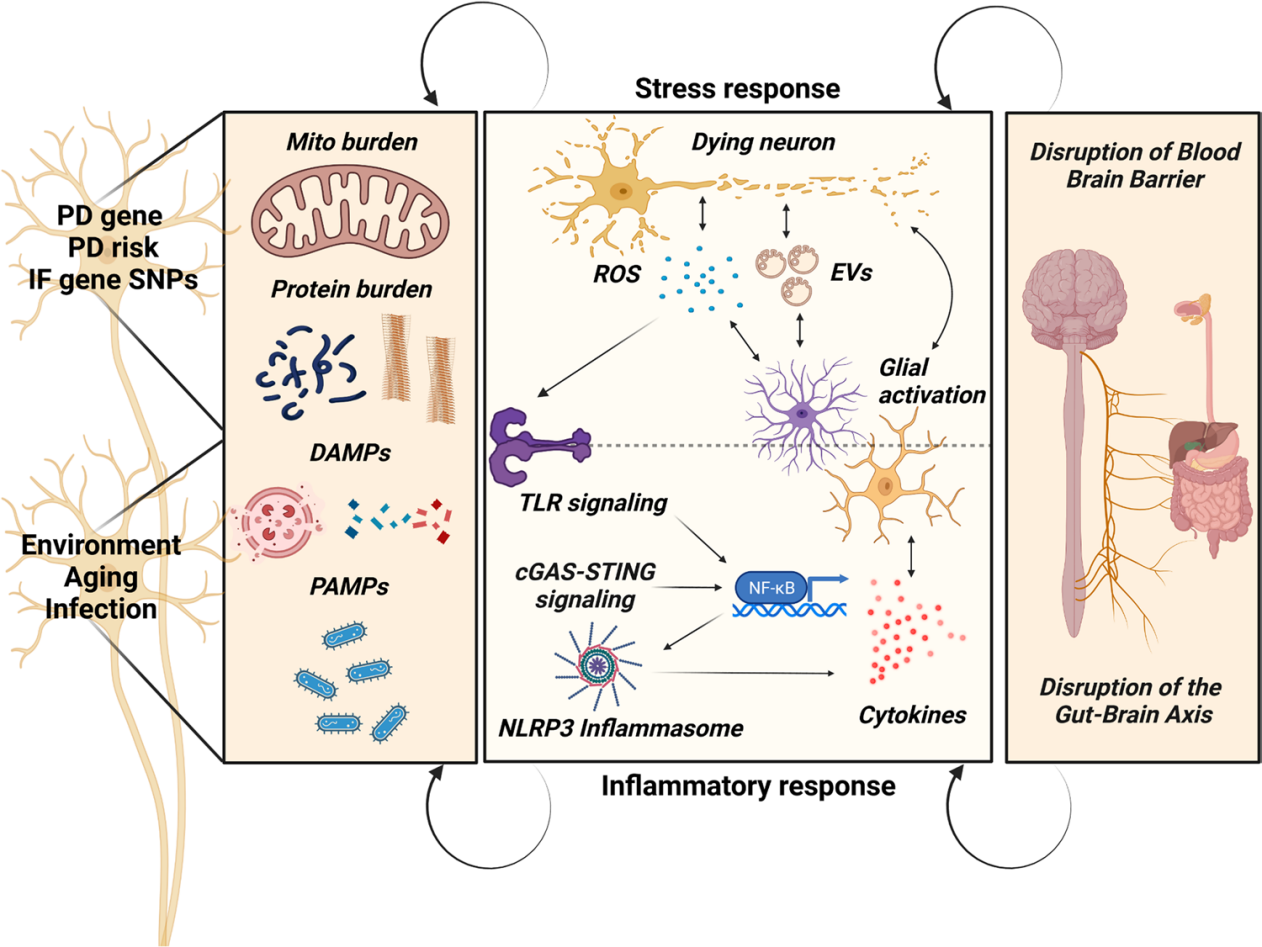
**Sustained loop of neurodegeneration and inflammation in Parkinson’s disease.** Parkinson’s disease (PD) gene mutations, PD-associated gene variants (PD risk), inflammatory gene single nucleotide polymorphisms (IF gene SNPs) plus environmental factors such as infection and aging can all trigger stress responses (upper, middle panel) and inflammatory responses (lower, middle panel) via mitochondrial damage (overall mitochondrial burden), protein burden (including impaired proteostasis but also impaired function) and the damage associated molecular patterns (DAMPs) as a result. Pathogen associated molecular patterns (PAMPs) also contribute to the triggering of stress and inflammatory responses. These self-supporting responses create a loop that facilitates further inflammatory signaling coupled with glial activation, production of reactive oxygen species (ROS) and changes to extracellular vesicle (EVs) release, which aggravates neuronal pathology. Disruption of the blood-brain barrier and gut-brain axis can further aggravate the self-sustained loop

The thin line between the physiological role of acute neuroinflammation and the pathological consequences of persistent neuroinflammatory responses is defined by the activity of multiple cell types involved in both innate and adaptive immunity [20]. These include not only the central nervous system (CNS)-resident glial cells (i.e. microglia, astrocytes and oligodendrocytes), but also peripheral circulating myeloid cells (i.e. monocytes, macrophages and dendritic cells) and T lymphocytes that actively participate to the neuroinflammatory process by infiltrating into the brain [21]. Mechanistically, following neuronal damage, the CNS-resident glial cells release signaling molecules (i.e. cytokines, chemokines, growth factors and other metabolites) that attract peripheral myeloid cells to the site of injury; in turn, myeloid cells have the ability to recruit other immune cells (T lymphocytes) within the CNS, thus amplifying the inflammatory response [22, 23]. Of note, not only glial cells, but also neurons can directly release inflammatory mediators that activate immune cells. This is a crucial notion to take into account to understand the temporal sequence of events (neurodegeneration vs neuroinflammation) that lead to irreversible cell death in PD. In fact, cell autonomous alterations inside SNpc DA neurons (e.g. mitochondrial dysfunction, defective protein clearance, α-synuclein release) can trigger, via the secretion of signaling molecules, an inflammatory response in the surrounding microenvironment or even at a systemic level, affecting other neuronal and non-neuronal cell types involved in the neuroinflammatory process [24•, 25] (Fig. 1).

### Environmental Triggers of Immune Response—the Gut Microbiome

It is worth noting that neuroinflammation-induced DA loss can be also triggered by peripheral non-cell autonomous mechanisms, as well illustrated by activation of the immune system in response to abnormalities in the gut microbiota [26]. A close connection between the CNS and the gut has been extensively described, the so-called gut-brain axis, and dysbiosis-induced inflammation could even represent the primary cause of neurodegeneration based on recent studies [6, 27•, 28, 29]. In PD, gastrointestinal symptoms such as constipation and increased gut membrane permeability often precede the motor symptoms, raising the possibility that gut inflammation triggered by a pathogen could play a role in the onset and progression of PD pathogenesis [30, 31].

If, on the one hand, brain can regulate gut activity through the parasympathetic and sympathetic nervous systems, by means of hormones and neurotransmitters that control digestive secretions and gut motility, on the other hand the gut microbiota also produces molecules able to influence activity of the host immune system and CNS function [32, 33]. Among these, pathogen-associated molecular patterns (PAMPs), including, e.g. bacterial lipopolysaccharides (LPS), peptidoglycans, CpG oligonucleotides and viral double-stranded RNAs (dsRNAs), can trigger the innate immune system by acting as ligands for the corresponding pattern recognition receptors (PRRs) on the surface of host cells [33]. PRRs are usually members of the Toll-like receptor (TLR) family, expressed on the plasma membrane of several cell types, including gut epithelial cells, myeloid cells, T lymphocytes, neurons and glial cells [34]. Under homeostatic conditions, a balanced gut microbiota results in a fine-tuned activation of TLR signaling in the gut epithelial cells, preventing induction of inflammatory pathways and maintaining the gut barrier intact. Conversely, modifications in the composition of microorganisms that populate the gut, also known as dysbiosis, may lead to dysregulated TLR signaling, persistent production of proinflammatory cytokines and disruption of the gut epithelial barrier. In turn, this results in a leakage of microbial-derived products and inflammatory mediators into the bloodstream, through which they can cross the blood-brain barrier (BBB) and finally reach the brain [34] (Fig. 1). Of note, in PD as well as in other brain diseases, neuroinflammation also dampens BBB integrity, making it more porous to substances, including toxins and bacterial metabolites, which may otherwise not be permitted through [35]. Other products of microbial metabolism, such as short-chain fatty acids (SCFAs), tryptophan metabolites and the amyloid *curli*, can have an impact of neuronal activity and modulate neuroinflammation [36–38]. Most SCFAs have anti-inflammatory properties (e.g. butyrate, valerate) and contribute to maintain the integrity of intestinal barrier; as a consequence, a low abundance of SCFA-producing bacteria may cause leakiness of the gut epithelium thus promoting the passage of intestinal pathogens into the bloodstream and systemic inflammation [39, 40]. Accordingly, reduced levels of the SCFA acetate, propionate and butyrate were detected in the stool of PD patients compared to age-matched controls and paralleled by a decreased amount of SCFA-producing bacteria, in particular from the families *Prevotellaceae* and *Lachnospiracae* [40, 41]. In support of these observations, transplantation of fecal microbiota from healthy donors as well as butyrate supplementation improved dopamine deficiency and motor impairment in different animal models of PD [38]. In parallel, we and others recently reported increased levels of *Lactobacillus, Christensenella* and *Akkermansia* in stool samples from PD patients [40]. Higher levels of Akkermansia could account for most of the metabolic alterations we predicted based on the different microbiota composition between healthy individuals and PD cases [41]. The neurotransmitter gamma-aminobutyric acid (GABA), the amino acid methionine and hydrogen sulfide (H_2_S) are the microbial metabolites with the highest predicted secretion potential we found [41]. To this regard, it is worth noting that GABA receptors have been found not only in the CNS but also in the ENS, where they could transduce signals regulating gastric motility and secretion. Importantly, GABAergic dysfunctions have been reported both in prodromal PD (mostly linked to gastrointestinal symptoms) and at later disease stages, but GABA contribution to neurodegeneration is still debated, as demonstrated by multiple studies showing controversial results [42, 43]. Interestingly, our study also highlighted a higher abundance of *Bifidobacteria* but only in constipated PD cases, thus suggesting a link between microbiota composition and intestinal non-motor symptoms in PD [41]. Of note, also pathogens that belong to the families *Enterococcaceae and Enterobacteriacee* significantly increased in fecal samples from PD patients, and this positively correlated with disease duration and severity [44, 45]. Last but not the least, *Helicobacter pylori* infection has been also associated to an increased risk to develop PD. Indeed, *H. pylori*-positive PD patients displayed higher mortality rate and motor dysfunction compared to healthy controls, a clinical phenotype significantly improved following *Helicobacter* eradication [46].

### The Role of Microglia

Different from macroglia (e.g. astrocytes, oligodendrocites), which arise from the neuroectoderm as do neurons, microglia arise from the peripheral mesoderm [13]. Microglia are the most relevant CNS-resident immune cells (mononuclear phagocytes) of the brain. While at rest, they are involved in synaptic pruning and remodeling thereby regulating brain connectivity [47]. By contrast, in case of a pathological insult, microglia change their morphology into an amoeboid shape and start to secrete inflammatory cytokines (activated state) [47]. Activated microglia engulf debris from surrounding cells such as degenerating dopaminergic neurons [47]. Accordingly, microgliosis is a typical histopathological hallmark of PD that was discovered as early as in 1988 [48]. The upregulation of proinflammatory enzymes and cytokines creates a microenvironment that is hostile for the survival of neurons [49, 50]. By contrast, it remains uncertain whether microgliosis is purely a consequence of neuronal demise in PD or whether microglial dysregulation can trigger neurodegeneration in the first place. In support of the latter hypothesis, PET studies using the 11C-(R)-PK11195 ligand that specifically binds to active microglia revealed that microgliosis can be observed during the early stages of the disease [51]. Recent advances in our understanding of the complex interplay between glia and neuronal cells in PD have come from single-cell studies [52, 53, 54, 55•]. Assessing the cellular diversity in murine brain tissue revealed an unexpected glial heterogeneity. Comparing mouse striatum and midbrain sections, the authors identified a microglia cluster with an “activated signature” specifically in the latter tissue. These cells overexpressed inflammatory genes as well as antigen presenting cell markers and shared transcriptional features of reactive microglia [53]. Recently, we also demonstrated, by means of single-nuclei sequencing, a specific upregulation of microglia in the midbrain tissue from sporadic PD (sPD) patients compared to controls [55•]. In particular, pseudo-time ordering identified an activation trajectory that branches out from resting microglia into two activated subpopulations—cells overexpressing *GPNMB* or such with high levels of *HSP90AA1* and *IL1B.* In addition, quantitative imaging analysis of sPD and control midbrain sections revealed a reduction in microglial branching exclusively in the *SN* of PD patients [55•]. Further strengthening the role of microglia in neurodegeneration, we observed an enrichment of PD risk variants specifically in these cells [55•]. Thereby the strongest association was detected for the PD-associated protein LRRK2, which has been intensively studied in the context of inflammation in PD as detailed in the coming sections. In light of the recent development of human microglial differentiation protocols, the findings from human post-mortem tissue can now be validated and extended in induced pluripotent stem cell (iPSC)-derived models.

### Evidence from Genetic PD

The inheritance of specific gene mutations in autosomal dominant (e.g. SNCA, LRRK2) and autosomal recessive (e.g. PINK1, PRKN, DJ-1) manner has been associated with the development of familial PD. PD caused by a known alteration to a single gene is collectively referred to as monogenic PD and accounts for about 10–15% of cases [56]. Most cases of PD have no known cause and are named ‘sporadic’ or ‘idiopathic’ PD (sPD). However, this terminology is currently being challenged, due to the involvement of gene variants or common genetic risk factors (e.g. GBA) in PD onset and progression [57]. The large body of mechanistic evidence suggesting that inflammation plays a significant role in PD pathogenesis has mostly come from studies of monogenic forms of the disease, where the emerging topics are aggregation or buildup of toxic proteins (including insoluble α-syn) in the brain as a trigger of inflammatory responses and wild-type PD proteins protecting dopaminergic neurons by suppressing inflammation in the surrounding cells.

#### SNCA

Duplication, triplication and point mutations in *SNCA*, the gene which codes for α-syn, are equally linked with late and early onset PD [58]. α-syn is a small protein capable of binding to membranes via its N-terminus. In the brain, the function of α-syn is to aid neurotransmission via the regulation of the synaptic vesicles and synaptic membranes [59]. In contrast, insoluble aggregates of α-syn, which are also a feature of sporadic PD, can trigger neuroinflammation [60]. Not only aggregation, but also the overexpression and post-translational modifications (e.g. nitration) of α-syn are linked to sustained microglial activation [61–66], an effect likely mediated by the NFkB and MAPK signaling pathways [64, 67]. Accordingly, the levels of neuroinflammatory markers, TNF, ICAM, IL-6 and IL-1α, are increased in the *SN* of mouse brain overexpressing α-syn [66]. Other studies have shown an activation of NLRP3 inflammasome in distinct α-syn PD models, including primary monocytes and primary microglia exposed to fibrillar α-syn, mouse striatum subjected to 6-OHDA treatment, α-syn pre-formed fibrils (PFF)-injected mice, post-mortem *SN* sections from PD brains and also blood and CSF samples from PD patients [68, 69, 70•]. Conversely, Piancone et al. recently showed that neither monomeric nor aggregated α-syn significantly activated the NLRP3 inflammasome in peripheral blood mononuclear cells (PBMCs) from PD patients[71]. Recent studies suggested that α-syn aggregation at nerve terminals as well as its capability to leave the cell into exosomes can trigger neuroinflammation and could be used as a potential biomarker for early PD diagnosis and a prognostic tool for disease progression [72, 73].

Interestingly, also α-syn silencing may cause neuroinflammation, suggesting that not only excessive α-syn concentrations but also perturbation of physiological α-syn levels could support PD pathogenesis [74].

#### LRRK2

Mutations in LRRK2 can cause autosomal dominant PD [75, 76]. LRRK2 is known to phosphorylate substrates in the endosomal-lysosomal pathway. The expression of LRRK2 is increased in monocytes activated by IFNγ, suggesting that it could play a role in monocyte maturation [77]. LPS-treated R1141G LRRK2 transgenic mice showed increased secretion of pro-inflammatory TNFα, IL-1b and IL-6, as well as a decreased expression of anti-inflammatory IL-10. mRNA levels of the pro-inflammatory cytokines TNF, IL-1, IL-12, CCL4, CXCL1, CCL3L1 were also increased, suggesting an upstream regulation by LRRK2 [78].

Higher LRRK2 kinase activity has been shown to be detrimental in normal cellular processes. The expression and kinase activity of LRRK2 were increased in LPS-treated lymphoblasts. LPS-dependent activation of TLR signaling pathway, increase in LRRK2/TRAF6 interaction and increased phosphorylation of both MAPK and IkB were reduced upon treatment with a LRRK2 inhibitor, leading to reduced TNFα secretion and thus confirming the involvement of LRRK2 in the generation of pro-inflammatory responses in lymphoblasts [79•].

#### PINK1 and Parkin

Mutations in the PARK6 gene encoding PINK1 can cause autosomal recessive PD [80]. PINK1 phosphorylates ubiquitin, regulates mitochondrial quality control and plays a key role in mitophagy (the removal of damaged mitochondria from cells). PINK1 is involved in the recruitment of cytosolic Parkin to damaged mitochondria [81]. Mutations in the PARK2 gene encoding Parkin also cause autosomal recessive PD [82]. Parkin is an E3-ubiquitin ligase that is responsible for the ubiquitylation and degradation of mitochondrial proteins and the recruitment of autophagy receptors that facilitate the removal of damaged mitochondria during mitophagy [83]. Parkin is known to regulate oxidative stress and maintain mitochondrial function within the cell and, together with PINK1, was originally shown to provide neuroprotection by activating the NFkB transcription factor that regulates immune response among others [84–87]. However, both increased and reduced activation of NFkB signaling have been observed in PINK1 KO mice and mouse macrophage cultures subjected to PINK1 knockdown [88, 89]. Parkin also suppresses inflammation by degrading TRAF2/6 and preventing the activation of NFkB and JNK signaling pathways [90]. Of note, many of these studies have shown that downregulation of either PINK1 or Parkin does not induce inflammation unless an external stressor like LPS or IFNγ is applied [85, 88, 91]. Several studies have suggested that wild type PINK1 inhibits peripheral inflammation [92], and both PINK1 and Parkin repress mitochondrial antigen presentation thereby preventing adaptive inflammation [93]. Accordingly, PINK1 deficiency triggers an innate immune response mediated by mitochondrial dysfunction and a dysregulation in anti-microbial defense mechanisms [94]. When subjected to exhaustive exercise, both PINK1- and Parkin-deficient mice trigger the pro-inflammatory cGAS-STING mediated Type-1 interferon response to foreign DNA [95•], resulting in increased serum levels of IL-6, IFN-β1, IL-12(p70), IL-13, CXCL1, CCL2 and CCL4 [96]. A similar response was observed in Parkin-KO mice genetically engineered to accumulate high levels of mtDNA mutations (PARK2^−/−^; mutator mice). Importantly, these mice display a motor deficit accompanied by the progressive loss of *SN* DA neurons with age, a phenotype rescued by the loss of STING [96]. Collectively, these findings revealed a functional link between mitophagy impairment caused by PINK1/Parkin loss and neurodegeneration in PD, which appears to be facilitated by systemic inflammation. Interestingly, reduction of STING pathway did not help to rescue PD phenotypes in Parkin-deficient *drosophila* models [97], highlighting that evidence for inflammation in PD is also subject to species differences. Since PINK1 only accumulates on depolarized mitochondria and substrates of the PINK1/Parkin pathway beyond mitophagy remain elusive, it is unclear by which mechanism PINK1 and Parkin inhibit inflammation under normal conditions. Recent proteomic studies have focused extensively on substrates of the PINK1-Parkin pathway [98], which does not suggest direct interaction with inflammatory proteins at least in human and mouse neurons during mitophagy. Perhaps, the integrity or quality of mitochondria rather than any direct action by PINK1 or Parkin is relevant for inflammation in PD. Underlying mitochondrial burden in sporadic PD may also play a role, involving small effect size variants in multiple nuclear-encoded mitochondrial genes affecting the same downstream molecular network (i.e. PINK1/Parkin-mediated mitophagy).

More recently, it was shown that intestinal infection/dysfunction in PINK1-KO mice and PINK1-mutant flies also leads to neuroinflammation and neurotoxicity [27•, 86]. These studies are important because until this point, researchers struggled to find significant Parkinson phenotypes in PINK1 or Parkin loss-of-function models in vivo. Also, several studies at that time showed that PINK1 and Parkin were not required for basal mitophagy nor in tissues of high energy demand in mice and flies [99–101]. These findings have reignited the discussion about whether PINK1 and Parkin could protect against inflammation via some form of evolved mitochondrial resistance. For example, Parkin was shown to mediate the response of mice to intracellular pathogens already a decade ago [102]. It is possible that the role of Parkin in mitochondrial clearance is tied to its suppression of inflammation via the mtROS-NLRP3 axis [103]. It is not known whether PINK1 (which is thought to act upstream of Parkin) is a necessity for evolved mitochondrial resistance or whether deficient PINK1-Parkin surveillance worsens mitochondrial dysfunction which then aggravates inflammation. The consensus is that the wild type PD proteins PINK1 and Parkin contribute to an overall inflammatory defense. Corti and others have gone further to suggest that the PINK1-Parkin mediated mitochondrial quality control in the glia is particularly important for protecting dopaminergic neurons [104].

#### DJ-1

Mutations in DJ-1 cause autosomal recessive PD [105]. DJ-1 has multiple neuroprotective functions, including the regulation of oxidative stress [106], but the link with inflammation is less clear. In Zebrafish, overexpression of DJ-1 in astrocytes was found to upregulate HMGB1 and Cyclophilin-A [107], which have both anti-inflammatory and pro-inflammatory functions. Proteomic analysis in the brain of DJ-1 knockout (KO) fish revealed downregulation of some inflammatory regulators, including the complement proteins c3a and c3b [108]. RNA sequencing analysis in the midbrain of DJ-1 KO mice also allowed to identify several genes potentially involved in neuroinflammation (e.g. Parp-1, Mmp8, Hmgn1, IL-1, Nfkbid), most of which were significantly upregulated [109•]. Recently, DJ-1 was shown to suppress NFkB signaling by directly interacting with its p65 subunit [110] and to mitigate inflammation mediated by the STING pathway [111]. Finally, a link between DJ-1 and regulation of gut microbiome was also described. In particular, DJ-1 KO mice display a significant increase in *Rikenella* and *Alistipes*, accompanied by higher levels of malonate in stool and serum, and elevated expression of fecal inflammatory proteins calprotectin and MCP-1. Importantly, expression of PD-related inflammatory genes in the midbrain of DJ-1 KO mice was significantly increased, suggesting a possible link between alteration of gut microbiome, metabolism and neuroinflammation in DJ-1 associated PD [109•].

#### GBA

Biallelic homozygous and compound heterozygous mutations in the GBA gene are known to cause Gaucher Disease, whereas individuals with heterozygous GBA mutations have a risk to develop PD which is 5 to 20 times higher compared to non-carriers [112, 113].

The GBA gene encodes Beta-glucocerebrosidase (GCase), which helps the breakdown of glucosylceramide into ceramide and glucose into lysosomes [114]. Knocking down GBA leads to α-syn aggregation, which in turn further impinges on GCase activity [115]. As described above, accumulation of α-syn itself can trigger a pro-inflammatory response; therefore, one can argue that inflammation observed in GBA-PD is simply mediated by α-syn pathology and not a direct consequence of GBA mutations. However, two independent studies carried out in mice carrying the homozygous GBA D409V mutation observed no inflammatory response, even in the presence of typical signs of α-syn pathology [116, 117]. In contrast, specific inhibition of GCase in mice resulted in α-syn neuropathology and inflammation, as revealed by elevated GFAP levels that are indicative of astrogliosis [118].

### The Importance of Intercellular Transmission

Based on recent studies, the cell-to-cell spreading of α-syn aggregates, and in particular the fibrillar forms of α-syn, can trigger an inflammatory response that contributes to PD progression [68, 119]. In fact, α-syn fibrils are able to induce the TLR2-NFkB signaling pathway in glial cells, resulting in microglia activation and release of proinflammatory cytokines [119, 120]. Several mechanisms have been proposed which may account for this α-syn spreading in a “prion-like” manner, including direct diffusion, exocytosis/endocytosis and uptake of EVs/exosomes [121–124]. In a very recent study, Heneka and colleagues showed that α-syn fibrils were readily taken up by microglial cells in a phagocytosis-dependent fashion and activate a transcriptional program related to pro-inflammatory and pro-apoptotic pathways [125•]. Interestingly, microglial cells were not able to promptly degrade α-syn aggregates but transferred α-syn to surrounding cells to reduce the degradation burden, which indeed resulted in improved α-syn clearance. The authors also demonstrated that, upon α-syn exposure, the number of intercellular microglial connections (i.e. tunneling nanotubes and gap junctions) dramatically increased, and this was accompanied by efficient transfer of α-syn aggregates from donor to acceptor cells [125•]. In particular, tunneling nanotubes (TNT) seem to play a crucial role in this process, as suggested by impaired α-syn transfer after treatment with a potent inhibitor of actin polymerization. Importantly, intercellular transfer via TNT also interested mitochondria, which are donated from healthy microglial cells to reduce inflammatory profile and cell death of dysfunctional, fibrillary α-syn-loaded, microglia. LRRK2 mutation impairs this ‘double-rescue’ strategy, as demonstrated by less efficient redistribution of α-syn between microglial cells from LRRK2 G2019S mice, thereby amplifying neuroinflammation [125•]. Finally, it is worth noting that transfer of fibrillary α-syn is not restricted to microglia but may also involve other cell types, such as astrocytes. To this regard, Rostami and colleagues recently showed in co-culture experiments that microglia can absorb and clear protein aggregates from the astrocytes [126], thus highlighting the importance of glial cell communication in the development of neuroinflammation and neurodegeneration in PD.

Extracellular vesicles (EVs) are small vesicles secreted by cells. Vesicles can arise from the budding of the endosomal membranes and can either fuse with the lysosome or with the plasma membrane and thereby be secreted into the extracellular space. Once in the extracellular space, they are commonly referred to as exosomes, which have a diameter of ~100nm [127]. EVs can circulate in the periphery (although the presence of neuronal EVs in blood is controversial) and have been shown to be taken up by other cells as a means of cell communication or sequestration of unwanted cargo. EVs are also involved in gene expression and contain non-coding RNA and proteins [127]. The possibility that EVs could harbor damaged or unwanted cargo from neurons in PD has led to extensive research into peripheral EVs as a potential window into the brain pathologic process [128] and as material for biomarker discovery in PD [129–131].

EVs contain both chromosomal and mitochondrial DNA, and recent work has shown distinct mitochondrial signatures in the EVs of PD patients [132]. Damaged or dysfunctional mitochondria are the main source of damage-associated molecular patterns (DAMPs), which play additional functions outside the organelle and are recognized by the immune system in a similar way to PAMPs (see above). They often stimulate an inflammatory response and any necessary regeneration. DAMPs have long been associated with dead and dying cells but can also arise following cellular stress. Mitochondrial DAMPs induce cytotoxicity, and cells have been shown to use the secretion of harmful molecules via EVs for cell homeostasis. Cytoplasmic release of damaged mitochondria DNA (mtDNA) is an important DAMP that initiates multiple inflammatory pathways, including STING, NLRP3 inflammasome and NFKB signaling [133]. MtDNA can also leave the cell (presumably via EVs) and cause damage to other cells and tissues. There is also a correlation between cell free mtDNA and neurological diseases with the presence of inflammation [134].

## Potential Biomarkers in PD Patient Cohorts

Starting from the 1990s, PD patient cohorts have provided biomaterials for inflammation studies on a large scale, thus allowing researchers to account for differences between individuals in the absence of isogenic controls. Early findings mainly looked at cytokines and other inflammatory markers in the cerebral spinal fluid (CSF) of PD patients, revealing increased levels of IL-1β and IL-6 [135, 136]. By the early 2000s, there were several genetic studies showing that inflammation could play a role in PD, as suggested for instance by the positive association with IL-1β polymorphisms [137, 138]. Based on growing evidence including the pioneering studies showing reactive microglia in the SN of PD patients [48], it has now been accepted that chronic inflammation in the basal ganglia of PD brains can contribute to the progressive loss of dopaminergic neurons.

Despite the early findings, few studies have convincingly shown increased inflammation in sporadic PD patients compared to healthy individuals [14, 139–141], and no inflammatory blood marker has been recommended as a potential biomarker for PD. So far, the most reliable biomarkers are limited to IL-1β and NLRP3 levels in genetic PD models. However, despite their significance, these innate immunity markers are also closely tied to the development of chronic diseases such as type 2 diabetes, obesity, retinal degeneration and other neurodegenerative diseases including Alzheimer’s disease and multiple sclerosis [142], suggesting that inflammation might only facilitate or aggravate PD pathogenesis rather than cause it. This is supported by work on T-cells isolated from PD patients, which are able to recognize α-syn peptides thus suggesting a role of adaptive immunity in PD [143]. Another study observed a defective meningeal lymphatic drainage in sporadic PD patients [144], known to impair clearance of toxic CSF waste from the brain leading to an aggravated inflammatory response [145].

Studies focused on either genetic risk or monogenic PD have supported the involvement of inflammation, but still no direct or unique inflammation biomarker for PD is evident that could be clinically translated. Increased plasma levels of IL-8, MCP-1, MIP-1-a, IL-1β and TNFα were shown to be associated with GBA-PD compared to sporadic PD [146, 147]. In a separate study, GBA mutation carriers without PD were found to have increased microglial activation compared to age matched healthy controls, suggesting that inflammation could be an early event. However, it cannot be predicted prematurely if the GBA mutation carriers will eventually develop PD. A thorough time course study in these carriers would be beneficial in correlating GBA mutations associated to inflammation to PD [148]. Plasma levels of ferritin, CCL18 and MIP1a were increased in PD patients with biallelic GBA mutations. Nevertheless, they were not increased in GBA-PD patients with heterozygous mutations suggesting that these markers are not promising to predict PD onset [149].

Serum of PD patients with biallelic PINK1 and Parkin mutations have high levels of IL-6 [24•, 96]. Increased circulating cell free levels of mtDNA (discussed as an inflammation trigger) were also observed in PD patients with PINK1 mutations. The study describes IL-6 as a PD state marker and circulating cell free mtDNA as a PD progression marker [24•]. Studies in the serum and cerebral spinal fluid (CSF) of asymptomatic LRRK2 mutation carriers, showing increased levels of IL-1β, PDGF, VEGF and IL-8 compared to healthy controls, have suggested these molecules as pre-clinical markers of PD onset and development [150]. Conversely, another study did not observe any difference in asymptomatic LRRK2 cohort and healthy controls arguing against inflammation as an early event in PD pathogenesis [151]. Interestingly, LRRK2 expression was upregulated in B cells, T cells and CD16 monocytes from sporadic PD patients compared to healthy controls [152], highlighting cell type specificity as an important factor in biomarker studies. Enrichment or separation of immune cells in blood could provide an opportunity to discover good biomarkers for PD even if the markers themselves are not cytokines or inflammatory proteins.

## Conclusions and Future Therapeutic Strategies

In developing useful treatments for PD, one major hurdle is the inconclusive and variable mechanistic data available, especially for sporadic forms of PD, which are difficult to model in vitro. Studies using patient-derived or isogenic iPSC-derived neuronal models as well as 3D brain organoids are still quite limited.

In this respect, focusing on patient-based biosamples and clinical data from large PD cohorts will be crucial for the identification and validation of novel and relevant biomarkers for different stages of PD and subsets of patients.

Overall, there is increasing evidence for the involvement of immune mechanisms and related inflammatory processes in the initiation and/or propagation of the neurodegenerative process in PD. Therefore, future neuroprotective treatment strategies need to account for these mechanistic insights, which are partially already reflected in available epidemiological data. In this context, an increasing body of evidence supported a protective role of anti-inflammatory medication in terms of risk to develop PD. It was shown that regular intake of nonsteroidal anti-inflammatory drugs (NSAID) was associated with a reduced risk for PD [153]. Interestingly, the LRRK2 gene, in which mutations are the most common cause of autosomal dominantly inherited PD, is encoding a protein that is highly expressed in immune cells, including blood cells and microglia. Therefore, it was speculated that the same LRRK2 gene that is also associated with inflammatory bowel disease [154] may modulate inflammatory pathways related to neurodegeneration. Recently, it was shown that indeed the LRRK2-related pro-inflammatory pathway can be modulated by regular NSAID intake. Here, it was shown that the penetrance of PD-linked LRRK2 mutations was reduced based on NSAID treatment [155]. These results may justify first clinical trials examining the potential modulatory effect of NSAID exposure on LRRK2-related PD.

An alternative potentially disease-modifying approach based on the increasing understanding of the role of the microbiome in PD pathogenesis relates to targeting the gut-brain-axis via acting on dysbiosis. It was shown that dysbiosis was associated with alterations of the protective mucus barrier within the gut and may favor the aggregation and propagation of the misfolded PD-associated protein alpha-synuclein [156, 157]. Therefore, interventions that may help to re-equilibrate the altered gut microbiota are currently investigated and include stool transplantation from healthy donors, which may prevent chronic inflammation and finally the pathology related to alpha-synuclein aggregation [156]. In mice, it was shown that indeed stool samples from PD patients can aggravate alpha-synuclein pathology and motor symptoms.

If alpha-synuclein pathology is not only triggered within the brain, but a more systemic degenerative process with contribution from chronic inflammation in the gut and mediated via the peripheral nerve system may play a role, systemic therapies targeting alpha-synuclein were further justified. Indeed, first clinical trials using antibodies raised against human alpha-synuclein are currently underway and may provide access to a causative treatment slowing disease progression in PD [159].

Future studies may integrate different pharmacological and lifestyle interventions aiming at positively modulating chronic inflammation and pathological alpha-synuclein aggregation as tightly interwoven processes in the pathogenesis of Parkinson’s disease and may focus on specific patient strata that can be defined genetically (e.g. LRRK2) or based on environmental factors (e.g. dysbiosis) and are enriched for the underlying chronic inflammatory process.
